# Optimization of Onion Peel Bioactive Compounds Using Eco‐Friendly Natural Deep Eutectic Solvent Based Ultrasound‐Assisted Extraction

**DOI:** 10.1002/fsn3.72339

**Published:** 2026-09-09

**Authors:** Izza Faiz Ul Rasool, Muhammad Umair Arshad, Ali Imran, Ayesha Siddiqa, Edouard Bugingo

**Affiliations:** ^1^ Department of Food Science, Faculty of Life Sciences Government College University Faisalabad Punjab Pakistan; ^2^ Institute of Food Science and Technology Khwaja Fareed University of Engineering and Information Technology Rahim Yar Khan Pakistan; ^3^ Department of Food Science and Technology, College of Science and Technology University of Rwanda Kigali Rwanda

**Keywords:** bioactive compounds, natural solvent, novel extraction, onion peel, waste valorization

## Abstract

The current study aimed to extract bioactive compounds from onion peel using ultrasound‐assisted extraction combined with natural deep eutectic solvents. Response surface methodology (RSM) design was applied to target the maximum extract yield of bioactive compounds. The extraction (UAE‐NADES) method was optimized using RSM design with varying parameters including water contents (A: 35%–55%), extraction time (B: 15–35 min), and solid‐to‐liquid ratio (C: 10–30 g/mL) as independent variables. Study findings showed that the highest total phenolic content (TPC) of 105.06 mg GAE/g and DPPH radical scavenging activity of 50.06 mg TE/g were observed highest at the 14th run [Conditions: Water: 35%, Extraction Time: 25 min, Solid‐to‐solvent ratio: 30 g/mL]. Comparison analysis of design runs also showed that factors like extraction time have a significant impact on phenolic content followed by water %. 3D response results also revealed that the set variables in the solvent mixtures significantly enhanced the extraction efficiency of phenolics and their antioxidant activities from source material (peel). Conclusively, study focused on emerging green extraction technologies for bioactive compounds recovery, gained lot of interest due to their efficiency as sustainable and environment‐friendly approach and for potential use in functional/nutraceutical food industries.

## Graphical abstract

This study focused on extraction optimization with natural deep eutectic solvents (NADES) applying Response Surface Methodology (RSM) design applied to target the maximum extract yield of bioactive compounds from onion peel ( *Allium cepa* L.). NADE solvent was used for the optimization. Results showed the higher phenolic contents and antioxidant activities were observed with Ultrasonic extraction at ultrasonic periods (25 min) and controlled water contents (35%) and Solid‐to‐solvent ratio (30 g/mL) with NADES (ChCl: CA). Polyphenolic compounds quantification through HPLC also support the premise that UAE is an efficient approach for bioactive compounds recovery, supporting the valorization of waste for industrial applications.

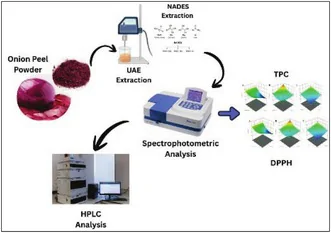

## 1 Introduction

The food processing industries generate large amounts of onion ( *Allium cepa* L.) waste particularly onion peel (> 1 million tonnes) often discarded in landfills, contributing to environmental pollution and waste management challenges (Jaganmohanrao 2025; Sajid et al. 2025). Organic waste of food industries are also considered serious waste burden if managed improperly. Onion by‐products include its peel, outer fleshy scales, onion bulbs and roots. Onion peel is valuable sources of bioactive phytochemicals (Joković et al. 2024; Halim et al. 2025), including phenolics, flavonoids, flavonols, anthocyanins, and polysaccharides (Senan et al. 2025; Ziaf et al. 2024). Due to its bioactive compounds, it can be used in different industries including food, pharmaceuticals, and cosmetics (Noman et al. 2025; Segundo et al. 2022).

*Allium cepa* L. is the second most cultivated vegetable with annual production of 100 million tonnes (Jaganmohanrao 2025). Commercial varieties of onion comprised on red, yellow and white onions which slightly differ in their chemical composition. Red onion peels generally have more chemical and nutritional composition as compared to the white and yellow onion peels. Quercetin and its glycosides are the most prevalent phenolic compounds in red onion (Senan et al. 2025), therefore, play a major role as antioxidant, antimicrobial and anticancer. By scavenging reactive oxygen species, controlling inflammatory pathways, and modifying cellular signaling, these phytochemicals demonstrate exceptional antioxidant, anti‐inflammatory, antibacterial, antiviral, antidiabetic, cardioprotective, neuroprotective, and anticancer properties (Celano et al. 2021; Kumar et al. 2022; Alawfi 2025). Onion peel high polyphenol and flavonoid content promotes a circular economy and waste valorization strategy in addition to providing potent antioxidant, antibacterial, and anti‐inflammatory properties (Maaz et al. 2025; Matra et al. 2025).

Ultrasound‐assisted extraction (UAE) is one of the emerging extraction technique widely employed for the extraction of bioactive compounds from various food and waste food materials (Siddiqa, Khaliq, Mehmood, et al. 2025; Tahir et al. 2024). Evidence reported UAE as an efficient extraction technique for extraction of bioactive compounds (Khalid et al. 2025), as it required less extraction time, low energy and safe (environment‐friendly). Recent research evidence reported natural and deep eutectic solvents (NADES) emerging green solvents to replace organic solvents. NADES are suitable for extraction due to nature of matrix; low temperature transition, low vapor and high solubility properties of donor and acceptor hydrogen bonds (HBD and HBA) (Sportiello et al. 2023; Wang et al. 2025).

In our study, the Response Surface Methodology (RSM) with Box‐Benhken Design (BBD model) was employed to maximize the extraction of bioactive compounds from red‐onion waste samples. Independent variables including UAE treatment time, water content, and solid‐to‐liquid ratio were tested (Levels: +1, −1) against objective variables. Current study was designed for valorization of onion waste as sustainable source of bioactive compounds. Green extraction solvents and the RSM design were employed for extraction optimization and for improved antioxidant activities of targeted bioactive compounds quantified from onion peel.

## 2 Material and Methods

### 2.1 Chemicals

All chemicals were purchased from Sigma‐Aldrich (USA). Other reagents employed were; Glycerol, citric acid, urea, choline chloride was used in extraction and sodium carbonate, ethanol (99%), methanol (99%), methanol, acetonitrile, Folin–Ciocalteu reagent, and 2,2‐diphenyl‐1‐picrylhydrazyl were used for quantification analysis.

### 2.2 Sample Preparation

Red onion peel samples (100 kg) were procured from the local market of Gujranwala, Pakistan. These onions are typically cultivated in the Punjab region under open‐field farming conditions and standard irrigation methods. However, the peels were manually sorted to remove damaged material, washed with distilled water to eliminate surface impurities, packed in sterile zip‐lock bags, and stored at ambient temperature until further processing.

Drying was carried out in a hot‐air oven at 50°C for 24 h to ensure complete dehydration and to minimize thermal degradation of bioactive compounds. The dried peels were then ground into a fine powder using a laboratory‐scale stainless steel electric grinder (The Cuisinart Spice & Nut Grinder‐Model SG‐10). The grinder has motor power ranged between 200 and 500 W and reduced particle size to < 500 μm. The powder was sieved through a 40–60 mesh sieve to obtain uniform particle size. After drying, onion peels were kept in sterilized zip lock plastic bags until further processing.

### 2.3 NADES Preparation

Natural solvents were formed by following the procedure of Khalid et al. (2025). The hydrogen bond acceptor (HBA) and the hydrogen bond donor (HBD) were combined according to the given molar ratio. Citric acid, urea, choline chloride, as well as glycerol were among the several chemicals used in the formulation of NADES, and the ChCl: CA, NADE solvent was used for the optimization. Solvent extractability comparison is provided in File S1. NADES were prepared by combining selected chemicals with defined molar ratio (1:1) (Table 1) and mixed using a magnetic stirrer to create a transparent, stable, and homogeneous mixture at 70°C. Then, NADES were placed in the dark and allowed to cool at room temperature.

**TABLE 1:** Composition of natural deep eutectic solvents.

NADES	HBA:HBD	Molar ratio
1	ChCl: CA	1:1
2	ChCl: Gly	1:1
3	ChCl: Ur	1:1

Abbreviations: CA, Citric acid; ChCl, Choline chloride; Gly, Glycerol; Ur, Urea.

### 2.4 Ultrasound Treatment

Onion peel samples were subjected to ultrasound treatment following the method of Khalid et al. (2025). Finely ground onion peel powder sample (50 mg) was mixed with solvent (3 mL) and subjected to ultrasound treatment in ultrasonic bath (UP200S, Hielscher, Teltow, Germany) at amplitude (40%), frequency of 40 kHz, and an ultrasound power of 200 W. After sonication, the sample was introduced into centrifugation (Tuttlingen, Baden‐Württemberg, Germany) for 15 min at 8000 rpm to separate the supernatant, and then was filtered.

### 2.5 Experimental Design

The Response Surface Methodology (RSM) design was employed to optimize the independent variables to evaluate their impact on dependent variables (Senan et al. 2025). Water content, ultrasonic extraction time, and solid‐to‐liquid ratio were selected as independent factors and investigated at three coded levels (−1, 0, +1) (Table 2). The experimental ranges varied from 35% to 55% for water content, 15–35 min for ultrasonic extraction time, and 10–30 (g/mL) for the solid‐to‐liquid ratio. These coded levels corresponded to actual values of 35%, 45%, and 55% water; 15, 25, and 35 min; and 10, 20, and 30 g/mL, respectively. Experimental data were analyzed using ANOVA to evaluate model significance, and Design‐Expert software (Version 13) was used to generate three‐dimensional response surface plots for visualization and optimization. All experiments were performed in triplicate to ensure reproducibility.

**TABLE 2:** Experimental ranges and levels for independent variables for response surface methodology design for NADES selection to optimize extraction.

Ind. variable	Symbols	Ranges and levels		
		−1	0	+1
Water content (%)	A	35	45	55
Ultrasonic time (min)	B	15	25	35
Solid/liquid ratio (g/mL)	C	10	20	30

### 2.6 Determination of Phenolics

Total phenolic contents of onion waste were determined using the Folin–Ciocalteu reagent assay method (Qin et al. 2026). The extract sample was measured as 20 μL mixed with 100 μL of Folin solution and 100 μL of sodium carbonate solution. After that, the mixture was put at room temperature for 1 h in the dark. The spectrophotometer was used to measure the absorbance at 765 nm, and the results are recorded as mg GAE/g dry weight.

### 2.7 DPPH Assay

The radical scavenging ability of onion extract samples was evaluated using the 2,2‐diphenyl‐1‐picrylhydrazyl (DPPH) assay method (Hanif et al. 2025). DPPH (4 mg) was dissolved in ethanol (100 mL), and onion peel extracts were measured (100 μL) in 96 wells of microplate. To speed up the reaction, the mixture was left in the dark for half an hour. A microplate reader was then used to measure the absorbance at 515 nm, and results were recorded as equivalents of trolox per g of dry weight.

### 2.8 High Performance Liquid Chromatography

High‐Performance Liquid Chromatography (HPLC) with photodiode array detector (PDA/DAD) and a Synergi Hydro‐RP C18 reversed‐phase column (250 × 4.6 mm, 4 μm particle size) (Phenomenex, Torrance, CA, USA). Column shielded by a C18 guard column was used for the analysis. Under gradient elution, solvents A (Milli‐Q water/acetonitrile, 95:5, v/v) and B (Milli‐Q water/acetonitrile, 50:50, v/v) made up the mobile phase. The injection volume was 25 μL, the flow rate was kept at 0.8 mL min^−1^, and the column was kept at 25°C following method (Qin et al. 2026). With a few minor adjustments, the validated HPLC‐DAD technique was used for the quantitative measurement of the targeted phenolics from onion extract samples (Ahmad et al. 2014). The amounts of each compound were quantified by measuring peak absorbance at different wavelengths (280, 313, and 450 λ) and retention time; results were represented as μg/g DW. Calibration curves of standards including ferulic acid, quercetin, gallic acid, caffeic acid, myricetin, kaempferol were drawn for compound identification in prepared samples. These compounds are known to be the main flavonoids in onion peel extracts made with green extraction methods such as NADES and UAE.

### 2.9 Statistical Analysis

In current study the dependent variables were analyzed using the Box–Behnken design for the process optimization. The mean ± standard deviation (±SD) was used to display the experimental data. For statistical computations, the Analysis of Variance (ANOVA) and Tukey's Test was applied using Minitab. To assess the mean differences, a new multiple‐range test was applied and for RSM design model and regression coefficient calculations were performed using Design Expert software Stat‐Ease 360 software version 22.0.3 (Stat‐Ease Inc., USA).

## 3 Result and Discussion

### 3.1 Optimization of Onion Peel Using NADES‐UAE

Three independent variables were assessed using the RSM based on the BBD in order to optimize the extraction conditions statistically. Independent variables were set including water content (A: 35%–55%), ultrasonic time (B: 15–35 min), and solid/liquid ratio (C: 10–30 g/mL). Table 3, presented variable factors and recorded responses at multiple runs (16 runs). Significant *p* values were defined as 0.0001 while *p* < 0.0001 was deemed significant in Duncan's test of significance. The NADES with the best extraction conditions were chosen following the screening study. Box–Behnken design (BBD) in conjunction with response surface methodology (RSM) was utilized for the NADES‐UAE process optimization in order to investigate the impact of UAE conditions on the extraction capacity of target compounds. Sixteen experimental runs according to the different combinations of NADES of different levels of independent variables described in Table 3.

**TABLE 3:** Independent factor and responses.

Runs	Factor 1	Factor 2	Factor 3	Response 1	Response 2
	A: Water	B: Time	C: Ratio	TPC	DPPH
	%	min	g/mL	mg GAE/g	mg TE/g
1	45	35	10	76.01	41.34
2	55	25	30	86.27	47.13
3	45	15	10	85.93	40.10
4	55	25	10	87.80	42.63
5	55	35	20	76.11	44.00
6	45	25	20	83.07	43.00
7	45	35	30	85.16	46.05
8	45	15	30	97.07	48.13
9	45	25	20	86.66	44.00
10	35	25	10	90.75	43.90
11	45	25	20	84.30	43.78
12	35	15	20	95.87	47.49
13	45	25	20	85.77	44.48
14	35	25	30	105.06	50.06
15	35	35	20	94.34	48.51
16	55	15	20	94.55	47.59

Abbreviations: DPPH, 2,2‐diphenyl‐1‐picrylhydrazyl; TPC, Total Phenolic Content.

Experimental and predicted values for independent variables (water content, sonication time, and solid to liquid ratio) and for phenolics and antioxidant (DPPH) was recorded as responses (Table 4).

**TABLE 4:** Predicted and experimental values at UAE optimal extraction conditions for onion peel samples.

UAE parameters			Values			
Water (%)	Liq./solid ratio (g/mL)	Time (min)	Responses	Predicted	Experimental	Error (%)
48.33	25.52	22.99	TPC (mg GAE/g)	87.30	87.01	0.33
50	25	23	DPPH (mg GAE/g)	45.41	46.11	1.54

Abbreviations: DPPH, 2,2‐diphenyl‐1‐picrylhydrazyl; TPC, Total Phenolic Content.

### 3.2 Total Phenolic Content (TPC)

**FIGURE 1:**
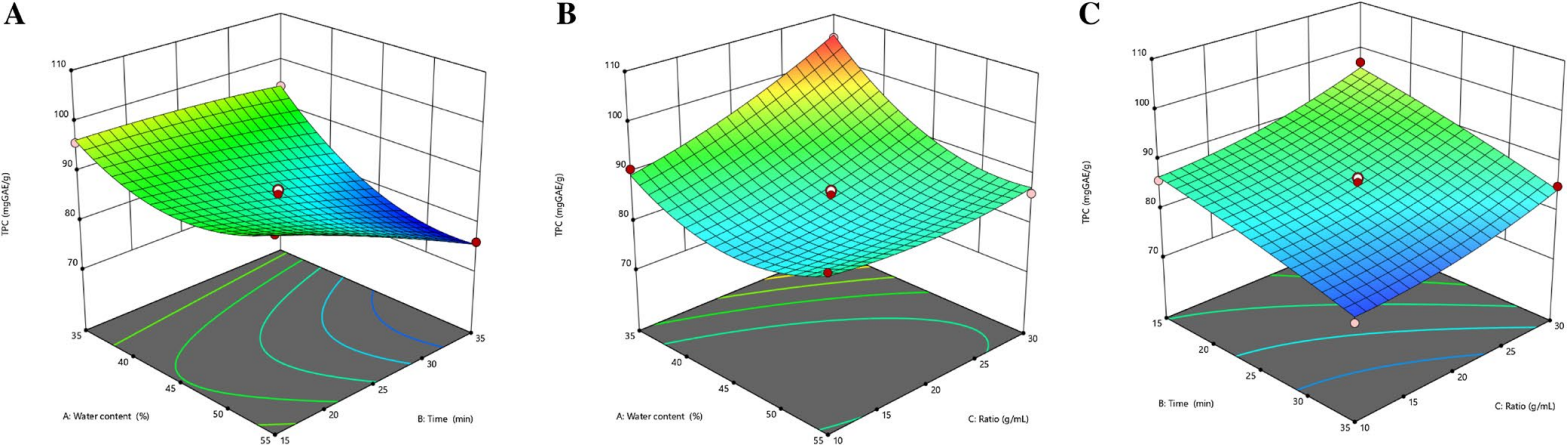
3D TPC interaction of water content and ratio.

Table 3 and Figure 1A present the results presenting the relationship between water content (A) and ultrasonic time (B), ranging from 76.01 to 105.06 mg GAE/g across the different treatments. Figure 1 illustrates with the increase in water content from a minimum of 35% and the time 25 min resulted in an increase in TPC to a maximum of 105 mg GAE/g. However, the TPC values dropped to 76 mg GAE/g at high water content (55%) at 35 min, most likely as a result of disintegration of phenolics when subjected to ultrasound for longer periods. The ideal range of extraction parameters and the gentle balance is needed to prevent overprocessing which in turn heats up the system which leads to dis‐intergradation (Bekavac et al. 2025). Overall, it is clear that the main factor affecting the extraction yield of TPC in the investigated experimental range is the water content of NADES followed by the extraction rate. This trend is consistent with the earlier research that examined the extraction of phenolic compounds utilizing NADES made of citric acid and choline chloride (Jovanović et al. 2022). Water (%) and liquid to solid ratio (mL/g) also showed a negative impact of higher water addition (Table 4). Results showed that an increase in water content (35%) and solid to liquid ratio (20 g/mL) of the system will increase the total phenolic content to 90 mg GAE/g. However, a further increase in water content (55%) and ratio (30 g/mL) resulted in a decrease in TPC to 85 mg GAE/g (Figure 1B). The results showed that interactions between water content and ratio were not significant (*p* > 0.0001). The regression model was used to analyze the effects of parameters and responses to evaluate the statistical significance of models (Equation 1).

(1) $$\text{Total Phenolic Content}\;\left(\mathrm{mg}\frac{\mathrm{GAE}}{g}\right)=84.95-5.16A\;p-5.22B+4.13C-4.23\mathrm{AB}-3.96\mathrm{AC}\;0.4975\mathrm{BC}+5.85A^{2}-0.5800B^{2}+1.67C^{2}$$

There is a strong correlation between independent factors and their responses recorded using the regression model equation and further confirmed by applying statistical analysis of variance. Hence, based on RSM data, the optimal conditions for TPC extraction from onion peel were; water of 35, time of 15 min, and ratio of 20 g/mL described in Table 5. According to the information currently available on NADES, physicochemical characteristics, their efficacy as extraction solvents is limited by their intrinsic high viscosity. Increased water content in NADES is recommended to improve extraction because it is linked to decreased viscosity, increased mass transfer, and easier diffusion in plant matrices (Teslić et al. 2022). It also increased the solubility rate of the target compounds, increasing the NADES extraction capacity (Moghimi and Roosta 2019). However, adding too much water can disrupt the network of intermolecular hydrogen bonds linked to NADES's solubility, which would reduce the extraction yield (da Silva et al. 2020).

**TABLE 5:** ANOVA for phenolic compounds of onion peel extract.

Source	Sum of square	df	Mean square	*F*‐value	*p*	
**Model**	852.73	9	94.75	36.36	0.0001	Significant
A‐Water content	213.11	1	213.11	81.78	0.0001	
B‐Time	218.40	1	218.40	83.81	< 0.0001	
C‐Ratio	136.70	1	136.70	52.46	0.0004	
AB	71.49	1	71.49	27.43	0.0019	
AC	62.73	1	62.73	24.07	0.0027	
BC	0.9900	1	0.9900	0.3799	0.5603	
A^2^	136.77	1	136.77	52.49	0.0004	
B^2^	1.35	1	1.35	0.5164	0.4994	
C^2^	11.19	1	11.19	4.29	0.0836	
Residual	15.64	6	2.61			
Lack of fit	8.08	3	2.69	1.07	0.4785	Nonsignificant
Pure error	7.55	3	2.52			
Cor total	868.36	15				
*R* ^2^	0.9820					
Adjusted *R* ^2^	0.9550					
Predicted *R* ^2^	0.8356					
Adeq precision	23.6023					
SD			1.61			
Mean			88.42			
C.V. %			1.83			

Conclusively, the best antioxidant recovery from onion skin was achieved with moderate ultrasonic periods (20–25 min) and controlled water contents (35%–45%). However, longer time or higher water percentage resulted in considerable TPC degradation. The best results for extracting polyphenols from vegetable peels were obtained when water concentration of solvent systems was between 30% and 40% (Prabawati et al. 2021). Results are in line with the previous study (Hammad et al. 2024) that investigated antioxidant potential employing UAE extraction with NADES confirmed that the increase in water content decreased mass transfer and solvent penetration, which may decrease the extraction efficiency.

In line with the findings, the statistical analysis and response surface plots showed interactions between the independent factors. The regression equation was solved and the 3D response surface plots are presented in Figure 1C. Mass transfer between the solvent along with sample is increased because it gives the acoustic waves greater surface area to create cavitation bubbles (Suleria et al. 2020). The increased exposure of the plant matrix to ultrasonic cavitation breaks down the cell wall and makes it easier for intracellular phenolic compounds (TPC ↑) to be released into the extraction media (Siddiqa, Khaliq, Sultan, et al. 2025). Mass transfer and solvent penetration are greatly influenced by the mechanical cavitation. However, prolonged ultra‐sonication caused free radicals that encourage the thermal and oxidative degradation of heat‐sensitive phenolic compounds (TPC ↓) (Bekavac et al. 2025).

Figure showed that longer initial extraction periods ensure that the sample is exposed to ultra‐sonication, which ruptures the cell wall and causes a more efficient release of intracellular components. However, because continuous ultra‐sonication creates rapid high temperatures, it may cause chemicals to degrade more quickly. The breakdown of bioactive substances can result from significant effects caused by an excessively high water content and ratio. First improved TPC by lowering solvent viscosity and boosting solvent diffusivity, increasing the water content in NADES. This facilitated mass transfer and increased the solvent's interaction with the phenolic‐rich onion peel matrix (Khalid et al. 2025).

The optimization of phenolic compounds extraction is highly dependent on processing parameters such as extraction time and solid to liquid ratio. As illustrated in Figure 1C, the total phenolic content (TPC) exhibits a significant response to variations in these two factors. Moderate treatment time (25 min) coupled with a maximum solid to liquid ratio (30 g/mL) resulted in the highest TPC yield.

This optimal condition likely enhances mass transfer efficiency, promoting the effective release of bound phenolic from the plant matrix into the solvent medium. The sufficient interaction time ensures that the phenolic diffuses efficiently without degradation or polymerization, while a high solvent volume provides an adequate medium for dissolution and dispersion of the extracted compounds. On the other hand, TPC significantly decreased with extreme treatment times (35 min) with lower solid‐to‐liquid ratios (10 g/mL) (Tul‐Muntaha et al. 2025). The total yield may be decreased by phenolic chemicals degrading or oxidizing as a result of prolonged exposure to processing conditions. Phenolic content may increase by lowering solvent viscosity and boosting solvent diffusivity, increasing the water content in NADES. This facilitated mass transfer and increased the solvent's interaction with the phenolic‐rich onion peel matrix (Senan et al. 2025). Water concentrations between 30% and 45% are generally regarded as advantageous because of increased phenolic solubilization by reducing viscosity while maintaining NADES's hydrogen‐bonding network. Excessive water addition impairs hydrogen‐bond interactions with target molecules, disturbs NADES's supramolecular structure, and may reduce cavitation efficiency, all of which lower extraction efficacy (Vo et al. 2024). Phenolic recovery was also greatly impacted by the solid‐to‐liquid ratio. Sufficient contact between the solvent and the plant matrix, a suitable concentration gradient, and improved diffusion of phenolic compounds were all made possible by an appropriate solvent volume (about 20 mL g^−1^). However, an overly large volume of solvent could dilute the extracted chemicals and decrease the ultrasonic energy density, which would limit the extraction efficiency and cavitation intensity (Zhou et al. 2024). Current study findings are consistent with previous research that reported limited solvent supply may limit extraction efficiency and that extended treatment can cause degradation of sensitive bioactive components. Thus, for TPC optimization, the relationship between treatment duration and solid‐to‐liquid ratio is essential. As a sustainable solution, to ensure higher yields of bioactive chemicals, process optimization is crucial to turn waste into a valuable resource, particularly for its application in food and pharmaceutical industries (Celano et al. 2021).

### 3.3 Radical Scavenging Activities of Onion Peel Extracts by Using DPPH Assay

**FIGURE 2:**
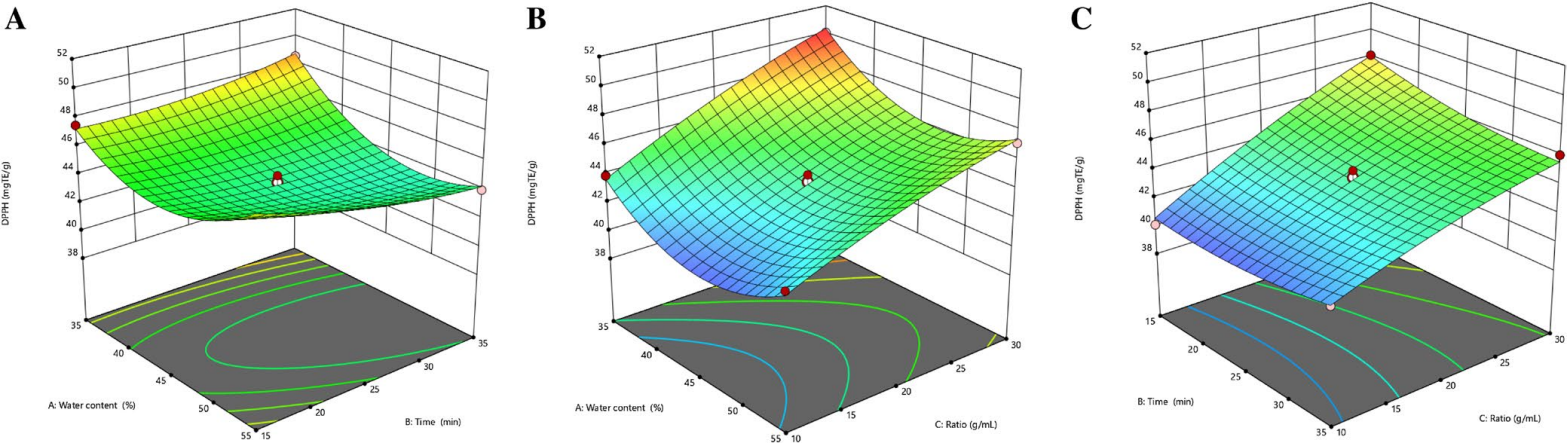
3D DPPH interaction of water content and time.

The DPPH assay results showed that the antioxidant activities are significantly affected by the interaction between water content and extraction period. A 3D response surface showing these two factors relate to DPPH radical scavenging activity is shown in Figure 2A. The highest DPPH radical scavenging activity was obtained at the lowest water content (35%) and a moderate extraction time (25 min), indicating that these conditions favored the extraction and preservation of antioxidant compounds. Moderate sonication provided sufficient disruption of the onion peel matrix to release antioxidants while minimizing their degradation. In contrast, the lowest DPPH activity (40.1) was observed at water content (45%) and with extraction period (15 min). These findings demonstrate that the interaction between water content and extraction time significantly influences antioxidant activities (Pal and Jadeja 2019), highlighting the importance of optimizing UAE conditions to maximize radical scavenging capacity (Table 6).

**TABLE 6:** ANOVA for DPPH assay for onion peel extract.

Source	Sum of square	df	Mean square	*F*‐value	*p*	
**Model**	115.90	9	12.88	37.46	0.0001	Significant
A‐Water content	9.27	1	9.27	26.96	0.0020	
B‐Time	1.45	1	1.45	4.23	0.0855	
C‐Ratio	68.44	1	68.44	199.13	< 0.0001	
AB	5.31	1	5.31	15.46	0.0077	
AC	0.6889	1	0.6889	2.00	0.2066	
BC	2.76	1	2.76	8.02	0.0299	
A^2^	26.09	1	26.09	75.89	0.0001	
B^2^	1.12	1	1.12	3.25	0.1213	
C^2^	0.7700	1	0.7700	2.24	0.1851	
Residual	2.06	6	0.3437			
Lack of fit	0.9204	3	0.3068	0.8060	0.5682	Nonsignificant
Pure error	1.14	3	0.3806			
Cor total	117.96	15				
*R* ^2^	0.9825					
Adjusted *R* ^2^	0.9563					
Predicted *R* ^2^	0.8579					
Adeq precision	21.0791					
SD			0.5863			
Mean			45.14			
C.V. %			1.30			

Furthermore, a shorter extraction period would restrict the release of bioactive substances and cell wall breakdown, which would reduce the extractor's capacity to scavenge radicals. This pattern indicates that although water is an essential solvent for the extraction of antioxidants, large amounts of it may prevent efficient recovery because of its limited solubility and greater polarity (Raza et al. 2025). The bioactive chemicals may not be completely released if the extraction time is too short, which could result in less than ideal antioxidant activity. Study results show the importance of extraction parameters especially time and solvent ratio in order to maximize antioxidant potential. The regression model used to analyze the effects of parameters and responses to evaluate statistical significance of models (Equation 2).

(2) $$\text{DPPH}\;\left(\mathrm{mg}\frac{\mathrm{TE}}{g}\;\mathrm{Dw}\right)=43.81-1.08A-0.426B+2.93C-1.15\mathrm{AB}-0.415\mathrm{AC}-0.8300\mathrm{BC}+2.55A^{2}+0.5288B^{2}-0.4387C^{2}$$

Solvents with a lower water content offer a higher extraction efficiency for bioactive chemicals that are moderately polar to nonpolar, including flavonoids and phenolic acids, due to solubilization and reduced polarity inconsistencies. High water concentration results in less hydrophobic antioxidant extraction because water is less effective than other solvents at dissolving less polar components and breaking down plant cell walls. Therefore, extraction time is considered critical for maximizing antioxidant potential.

The DPPH radical scavenging activity is determined by the interplay between the solid‐to‐liquid ratio and the water content, as seen in Figure 2B. This 3D response surface shows how these characteristics have a significant impact on antioxidant potential. With the highest solid to liquid ratio (30 g/mL) and the lowest water content (35%), the maximum DPPH value was achieved.

Effective mass transfer made possible by the greater solvent availability increases the phenolic compounds' ability to scavenge radicals. On the other hand, the lowest solid‐to‐liquid ratio (10 g/mL) and the medium water content (45%) displayed the lowest DPPH activity. The concentration of antioxidant molecules and their overall ability to neutralize DPPH radicals may be reduced by high water content. Additionally, because there is less solvent available to dissolve and disperse bioactive compounds, a lower solid‐to‐liquid ratio results in less than ideal recovery and decreased extraction efficiency. The negative relationship between water content and the solid‐to‐liquid ratio emphasizes the careful balance needed to maximize antioxidant extraction (Koraqi et al. 2024).

The results indicate that the best way to maximize antioxidant activity in plant‐based extracts is to increase the solid‐to‐liquid ratio while keeping the water content lower. These findings offer insightful new information about how to best optimize the extraction conditions for formulations high in antioxidants. However, the lowest antioxidant activity was observed at medium water content (45%) and the lowest solid‐to‐liquid ratio (10 g/mL). A higher water content tends to dilute bioactive compounds, whereas a low solid‐to‐liquid ratio reduces the effective concentration of the solute in the solvent, lowering the driving force needed for mass transfer (Yeler and Nas 2020).

As illustrated in Figure 2C, the DPPH radical scavenging activity is significantly influenced by the solid‐to‐liquid ratio as well as extraction time. The 3D response surface illustrates the impact of altering these two factors on plant extracts' antioxidant qualities. The lowest solid‐to‐liquid ratio of 10 g/mL and a moderate extraction duration of 25 min produced the greatest DPPH value. This shows that a shorter solid‐to‐liquid ratio avoids excessive dilution and preserves a higher concentration of recovered bioactive molecules, while a sufficient processing time permits the effective release of antioxidant chemicals. However, there is a noticeable reduction in DPPH activity as the extraction duration and solid‐to‐liquid ratio keep decreasing. The lowest solid‐to‐liquid ratio (10 g/mL) and the shortest extraction period (15 min) yield the lowest DPPH results. Additionally, by preventing phenolic and flavonoid compounds from being soluble in the extraction medium, a low solvent‐to‐solid ratio may reduce the mass transfer efficiency (Lee et al. 2024). The diffusion and solubilization of antioxidants into the solvent phase are facilitated by a lower solid‐to‐liquid ratio (10 g/mL), which may increase solvent accessibility to the plant matrix by offering a greater solvent volume per g of solid. To maximize the extraction yield of phenolic compounds and improve their antioxidant activities, the choice of the right solvent and its volume (liquid to solid ratio) matrix is crucial. To create effective extraction procedures for functional foods and nutraceutical applications, it is essential to comprehend dependent factor interactions.

### 3.4 High Performance Liquid Chromatography (HPLC)

**FIGURE 3:**
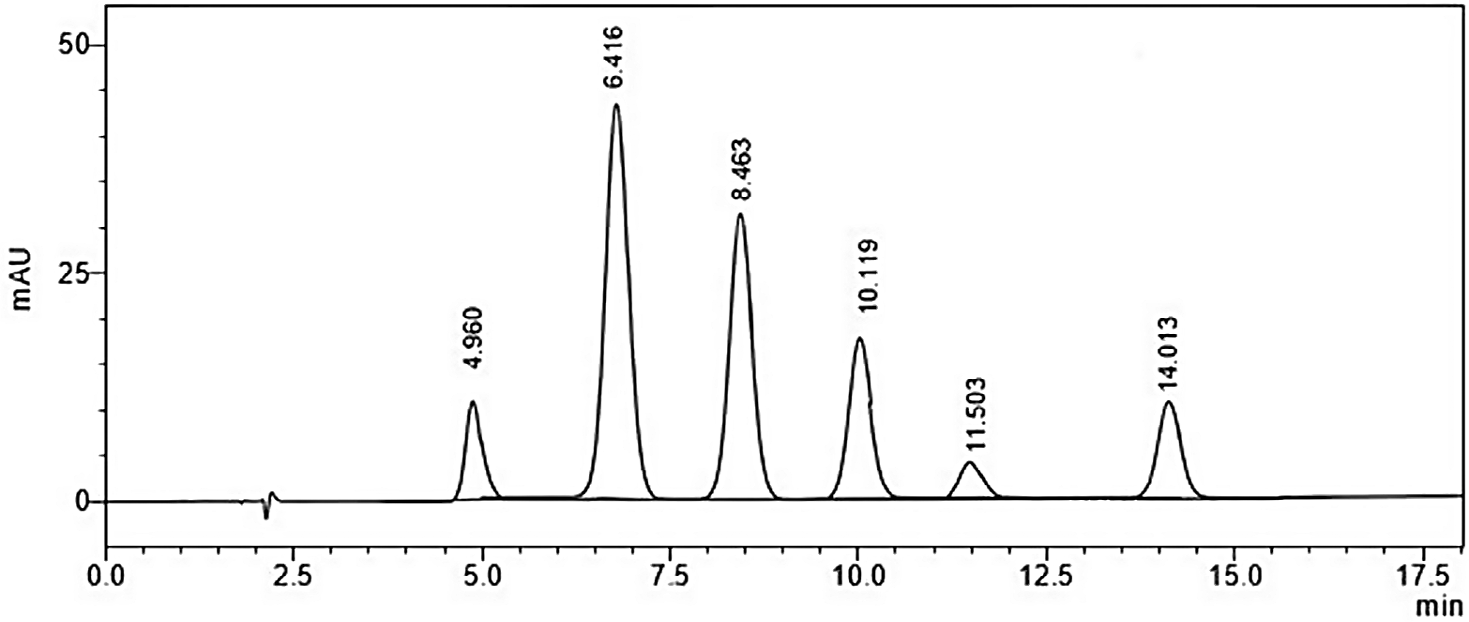
Peaks identification of red onion peel extract sample.

High‐performance liquid chromatography (HPLC) is an effective analytical method widely used for separation, identification and quantification of bioactive compounds in complex food matrices. Onion peel sample extract prepared at optimal conditions (Run 14) was subjected to identification and quantification of bioactive compounds through HPLC. Results showed different bioactive compounds from various polyphenol classes including phenolics and flavonoids. The concentration of onion peel powder was measured in mg per 100 g. Presented results (Table 7) showed different bioactive compounds identified at different retention periods: Ferulic acid [RT: 4.96 min], Gallic acid [RT: 6.41 min], Caffeic acid [RT: 10.11 min], Quercitin [RT: 6.41 min], Myricetin [RT: 11.50 min], and Kaempferol [14.01 min] (Figure 3). Among various bioactive compounds, the highest concentration 5.097 mg/g DW was observed for quercitin followed by gallic acid and caffeic acid (Figure 3). Current study results are supported by previous research database reported Yemeni red onion peel extracts evaluated for polyphenolics and found phenolic acids, flavonoids and other phenolic derivates. Phenolic acids including gallic acid (1.50 mg/g DW), syringic acid (5.19 mg/g DW), chlorogenic acid (1.87 mg/g DW) and flavonoids: quercitin (5.22 mg/g DW), myricetin and kaempferol (0.130 mg/g DW) were found in onion peel extracts by HPLC (Senan et al. 2025). In another research study, or standard polyphenols, namely quercetin, its dimers and trimers high concentration was found in onion skin residues (Gomes et al. 2020). In another study, major flavonoids were focused in onion peel NADES extracts and found significant concentration of flavonoids [Quercetin 6.18 mg/g, kaempferol 0.34 mg/g, and myricetin 0.15 mg/g DW] (Pal and Jadeja 2019).

**TABLE 7:** Identification and quantification of HPLC identified polyphenols at various retention periods.

Retention time (min)	Compound name	mg/g DW
4.960	Ferulic acid	0.165
6.416	Quercetin	5.097
8.483	Gallic Acid	1.070
10.119	Caffeic acid	0.503
11.503	Myricetin	0.120
14.013	Kaempferol	0.145

## 4 Conclusion

In the study, extraction process optimization was successfully achieved by using UAE combined with NADES from onion waste (peel). Optimization with RSM and BBD was targeted for extraction of bioactive compounds and radical scavenging activities. The optimal UAE–NADES extraction conditions were 35% water content, 25 min extraction time, and a 30 g/mL solid‐to‐liquid ratio, yielding highest total phenolic content (105.06 mg GAE/g) and DPPH radical scavenging activity (50.06 mg TE/g). However, chromatographic (HPLC) analysis confirmed polyphenolic compounds for phenolic acids (Gallic acid 1.07 mg GAE/g) and flavonoids (Quercitin 5 mg/g Dw) majorly found in red onion peel extract. The results demonstrate that onion peel, an abundant agro‐industrial by‐product, can be effectively transformed into a functional ingredient suitable for food and pharmaceutical applications, supporting sustainable waste utilization and circular bio‐economy development. Future studies require investigation of environmental impact through Life cycle assessment (LCA), and in vivo digestion to confirm cell line gastrointestinal tract (GIT) for food and nutraceutical applications.

## Author Contributions

**Izza Faiz Ul Rasool:** writing – review and editing, conceptualization, formal analysis. **Muhammad Umair Arshad:** writing – review and editing, methodology. **Ali Imran:** writing – review and editing, supervision. **Ayesha Siddiqa:** writing – review and editing, formal analysis. **Edouard Bugingo:** writing – review and editing, conceptualization.

## Conflicts of Interest

The authors declare no conflicts of interest.

## Supporting information


**File S1:** fsn372339‐sup‐0001‐FileS1.docx.

## Data Availability

The data that support the findings of this study are available from the corresponding author upon reasonable request.
