# Supplementary material for: Optimization of Onion Peel Bioactive Compounds Using Eco‐Friendly Natural Deep Eutectic Solvent Based Ultrasound‐Assisted Extraction
Source: Food Sci Nutr. 2026 Sep 9;14(9):e72339. doi: 10.1002/fsn3.72339 (PMC13555398; doi:10.1002/fsn3.72339)
Supplement: Supplementary file 1 [file FSN3-14-e72339-s001.docx]

**Supplementary Data:**

**Response to reviewer comment:**

**Comment:** What are the scientific backgrounds for the determination of experimental parameter ranges for optimizations such as (10-30 g/mL, 35-45% of water content, and 15-35 minutes of ultrasonication time) without single-factor screening experiments?

**Response:**

Single-factor screening experiments were performed for the determination of experimental parameter ranges for optimizations.

| Ratio | TPC mgGAE/g |
| --- | --- |
| 1:10:00 | 70.08 |
| 1:20:00 | 81.75 |
| 1:30:00 | 77.34 |
| 1:40:00 | 65.56 |
| 1:50:00 | 55.79 |

| Time | TPC mgGAE/g |
| --- | --- |
| 5 | 69.65 |
| 15 | 80.43 |
| 25 | 84.23 |
| 35 | 79.12 |
| 45 | 65.98 |
| 55 | 63.06 |

| Water | TPC mgGAE/g |
| --- | --- |
| 15 | 45.98 |
| 25 | 69.13 |
| 35 | 82.34 |
| 45 | 90.03 |
| 55 | 80.77 |
| 65 | 59.54 |

**Comment: Which NADES was used for the optimization? Please confirm it.**

**ChCl: CA,** NADE solvent was used for the optimization. Solvent extractability comparison is provided in supplementary file.

| Solvent | mgGAE/g |
| --- | --- |
| Water (100%) | 45.89 |
| Ethanol:Water (50:50) | 62.09 |
| Methanol:water (50:50) | 66.23 |
| ChCl: CA | 84.64 |
| ChCl: Gly | 76.11 |
| ChCl: Ur | 71.09 |

**Solvent Comparison for Optimal Extraction**
